# Comparison of Antimicrobial Resistance, Virulence Genes, Phylogroups, and Biofilm Formation of *Escherichia coli* Isolated From Intensive Farming and Free-Range Sheep

**DOI:** 10.3389/fmicb.2021.699927

**Published:** 2021-07-30

**Authors:** Xueliang Zhao, Yunyang Lv, Fathalrhman Eisa Addoma Adam, Qingfang Xie, Bin Wang, Xindong Bai, Xiaoyuan Wang, Honghu Shan, Xinglong Wang, Haijin Liu, Ruyi Dang, Juan Wang, Zengqi Yang

**Affiliations:** College of Veterinary Medicine, Northwest A&F University, Yangling, China

**Keywords:** antimicrobial resistance, biofilm, *Escherichia coli*, phylogenetic groups, virulence gene, zoonotic potential

## Abstract

Pathogenic *E. coli* are among the most frequently isolated bacterial pathogens on large-scale sheep farms in China. Antibiotic use in wool sheep production is a risk factor for promoting the emergence of resistant *E. coli*. To reveal the differences of *E. coli* populations in sheep from different farming systems the antimicrobial resistance, virulence genes, biofilm formation, and phylogroups of 500 *E. coli* isolates obtained between September 2019 and December 2020 in northwest China from diarrheic infections of intensive farming and free-range sheep were analyzed. The antimicrobial susceptibility test for 12 classes of antimicrobial agents was determined using the broth microdilution susceptibility method, and PCR was used to detect the differences in virulence genes and phylogroups. Additionally, biofilm formation was determined using microtiter plate and slide agglutination methods. Among the 500 *E. coli* isolates, the majority of the isolates were multidrug resistant (75.4%) and carried at least one virulence gene (94.8%). We observed that 412 (82.4%), 360 (72.0%), and 266 (53.2%) are found to be resistant to sulfisoxazole, florfenicol, and tetracyclines, respectively. Resistance was also observed to mequindox (46.8%), ampicillin (43.6%), spectinomycin (38.6%), enrofloxacin (34.2%), ceftiofur (21.0%), gentamycin (20.4%), ceftazidime (17.8%), and polymyxin B (7.8%) but no resistance was found to meropenem. These results showed that strains from free-range subjects had fewer antibiotic resistance strains rather than sheep that were intensively farmed (*P* < 0.05). We observed fifteen virulence genes, of which *etrA* (*n* = 401, 80.2%) is the most common. In addition, EAEC (86.4%) is dominant among free-range sheep and EHEC (80.1%) is dominant among intensive farming. Among all virulence genes, the strongest correlation was found between *etrA* and *papC* gene (*P* < 0.001, OR = 455.68). Similarly, the strongest correlation was also found between *eltA* and sulfisoxazole (*P* < 0.001, OR = 877). Furthermore, the majority of the *E. coli* isolates belonged to phylogroup B1 (50.6%), followed by phylogroup C (20.6%), A (7.4%), E (7.4%), D (5.8%), B2 (1.6%), and F (1%). Interestingly, phylogroup B2 and D were all distributed in intensive farms. In addition, 33 (6.6%), 373 (74.6%), and 94 (18.8%) showed moderate, weak, and no connection biofilm formation ability, respectively. These data uncovered that wool sheep serve as a reservoir of pathogenic *E. coli* harboring multiple resistance phenotypes and virulence genes. The overlapping virulence-associated traits between IPEC and ExPEC indicated the zoonotic potential and safety threats of sheep food products. It is urgent to improve the proper use of antimicrobials in China as well as other countries.

## Introduction

*Escherichia coli* (*E. coli*) is one of the most important bacteria for animals and humans. It is distributed all over the world and will bring huge economic losses to the breeding industry and public health. Pathogenic *E. coli* can produce potent toxins and is divided into intestinal pathogenic *E. coli* (IPEC) and extraintestinal pathogenic *E. coli* (ExPEC) according to the production of virulence factors (such as adhesin, capsule synthesis, toxins, etc.) (Habouria et al., 2019). The IPEC is subclassified into Enteropathogenic *E. coli* (EPEC), Enterotoxigenic *E. coli* (ETEC), Enteroaggregative *E. coli* (EAEC), Enteroinvasive *E. coli* (EIEC), and Enterohemorrhagic *E. coli* (EHEC) (Guimarães et al., 2019). IPEC infection can cause diarrhea, intestinal inflammation, and even deaths in severely affected animals and children (Malberg Tetzschner et al., 2020). In addition, ExPEC is mainly responsible for urinary tract infections (UTI) and is the main cause of meningitis, especially for babies, within 30 days of birth (Russo and Johnson, 2003).

In China, *E. coli* is ubiquitously distributed in the whole country with variable prevalence between intensive farming and free-range wool sheep. Current control strategies against *E. coli* mainly rely on repeated antibiotic treatments. However, in the last 20 years, the widespread use of antibiotics, including misuse and overuse, has helped bacteria naturally evolve and develop resistance (Gabale et al., 2020). Meanwhile, continuous use of antibiotics in animals strengthens the selective pressure and the onset of drug-resistant strains, which are easily transmitted to humans (Laxminarayan et al., 2013; Van Boeckel et al., 2017). Pathogenic *E. coli* of wool sheep can be used as a major reservoir for drug-resistant genes and the spread of resistant bacteria from food animals to humans through the food chain. The British government report estimates that by 2050, the number of deaths due to antibiotic-resistant infections will exceed 10 million every year (O’Neill, 2016). Each year in Europe, about 25,000 people die and up to 2.8 million people get a drug-resistance bacterial infection (ECDC/EMEA, 2009). As such, one of the greatest threats to humanity in the present world is the potential emergence and global spread of resistant bacteria (Nadeem et al., 2020).

Ningxia, Shaanxi, Inner Mongolia, and Qinghai are all major animal husbandry provinces in China. However, in recent years, antimicrobial-resistant *E. coli*, especially multiple drug-resistant (MDR) strains have caused frequent outbreaks of fatal hemorrhagic diarrhea diseases in wool sheep. Wool sheep are one of the most important reservoirs for pathogenic *E. coli* that can cause a series of clinical symptoms (Wani et al., 2007; Kim et al., 2010). As such, it is necessary to explore the antimicrobial resistance, virulence genes, biofilm formation, and phylogenetic groups of *E. coli*, which are critical for the therapeutic management of sheep. Whether in human or veterinary medicine, previous reports on bacterial isolates have revealed the relationship between virulence factors and resistance genes (Pan et al., 2020; Pérez et al., 2020). However, despite several studies, these links remain unclear. In addition, these studies evaluated the resistance of *E. coli* isolates from animals, rather than the resistance of strains derived from different feeding methods.

It is therefore currently necessary to assess the risk of *E. coli* antibiotic resistance (AMR) and virulence genes in sheep for public health. However, little is known about antimicrobial resistance, virulence factors, biofilm, and phylogenetic groups of wool sheep *E. coli* isolated in China (Chandran and Mazumder, 2013). Therefore, the detection of virulence genes and the analysis of drug resistance in wool sheep *E. coli* have significance for the prevention and control of diseases caused by *E. coli*. Furthermore, further research in this area will be beneficial for minimizing the increasing transmission of drug-resistant *E. coli* strains in the wool sheep. To our knowledge, this is the first report on antibiotic resistance, virulence factors, and phylogroups, characteristics in *E. coli* strains isolated from wool sheep with diarrhea that compares intensive and free-range farming in China.

## Materials and Methods

### Bacterial Isolation and Identification

**FIGURE 1:**
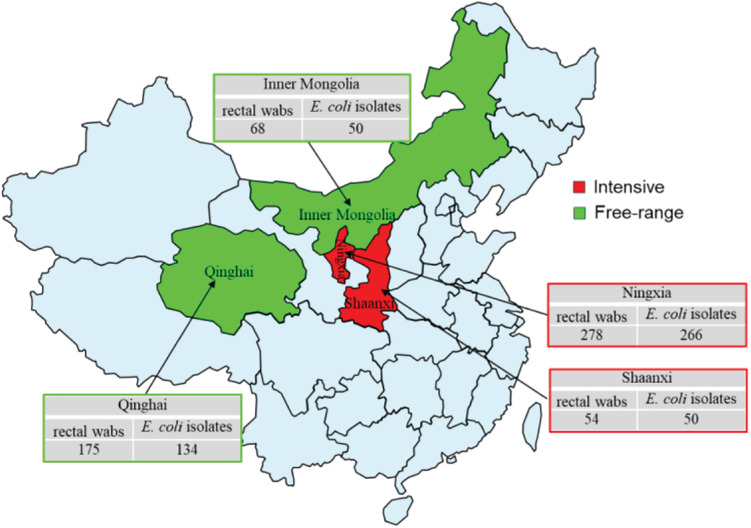
Sample collection regions in China during 2019–2020. The rectal swabs were collected from intensive farming wool sheep with diarrhea, Ningxia (East longitude 103°40′, Northerly latitudes 36°03′), Shaanxi (East longitude 109°49′, Northerly latitudes 34°45′), free-range wool sheep with diarrhea, Qinghai (East longitude 101°77′, Northerly latitudes 36°62′), and Inner Mongolia (East longitude 109°99′, Northerly latitudes 39°82′). Red and green colors indicate intensive and free-range farming, respectively.

A total of 575 rectal swabs were collected from adult wool sheep with diarrhea, in Northwest China, between September 2019 and December 2020. Figure 1 illustrates the geographical region where the rectal swabs were collected, which includes the locations of Ningxia, Shaanxi, Qinghai, and Inner Mongolia. All rectal swabs were transported to *Northwest A&F University* (NWAFU Yangling, China), College of Veterinary Medicine, Microbiology, and Molecular Diagnostic laboratory under freezing conditions within 2 h. The Animal Welfare and Research Ethics Committee of Northwest A&F University approved the protocol of the experiment (Number: NWAFUSM2019009). This study was performed under standard biosecurity and institutional safety procedures. Rectal sterile swabs were used to isolate and identify *E. coli*, and only one isolate was examined for each sample. The rectal swab of each sample was inoculated into sterile tubes containing 10 ml of Luria-Bertani (LB) broth and mixed vigorously (180 r/min) overnight at 37°C. After enrichment, a loopful of the broth was streaked onto MacConkey agar (Oxiod) and incubated aerobically at 37°C for 18–24 h. From each plate, a single pink-colored sample was chosen, and sub-cultured onto eosin-methylene blue (EMB) agar (Oxiod), before being incubated as described above. Colonies showing greenish metallic colors were considered presumptive *E. coli* isolates, and then confirmed as *E. coli* via biochemical analysis, using Biomerieux VITEK2 Compact Automated microbial identification system (BioMerieux, France), according to the manufacturers. The *E. coli* strains were suspended in 20% glycerol broth at −80°C for further bacteriological analysis.

### Antibiotic Sensitivity Testing

The antimicrobial susceptibility was tested by the broth microdilution method using Mueller-Hinton agar (Oxoid) as a culture medium according to the Clinical and Laboratory Standard Institute (CLSI VET08., 2018; CLSI; Wayne PA., 2020) guidelines as has been previously described. The minimum inhibitory concentrations (MIC) of each antibiotic was classified as resistant (R), intermediate (I), or susceptible (S) based on the CLSI breakpoints when available; otherwise, the National Antibiotic Resistance Monitoring System, NARMS^1^ for intestinal bacterial breakpoints was used. The susceptibility of *E. coli* was tested for 12 antibiotics: sulfisoxazole, spectinomycin, mequindox, ampicillin, gentamycin, tetracyclines, florfenicol, ceftazidime, ceftiofur, meropenem, polymyxin B, and enrofloxacin. When proven to be resistant to three or more antibacterial agents, *E. coli* isolates are considered to be MDR (Exner et al., 2017). The test was done in triplicate for each strain and *E. coli* ATCC 25922 (China Institute of Veterinary Drug Control) as a quality control strain. Based on the results of MIC, 500 strains of *E. coli* were screened for subsequent resistance genes, virulence genes, phylogenetic grouping, biofilm formation, and serological identification.

### DNA Extraction and Virulence Factors (VFs) Determination

A single colony of a fresh bacterial culture from LB solid medium was picked and resuspended in 150 μl of sterile ddH_2_O. Tubes were heated at 100°C for 10 min and subsequently centrifuged at 12,000 rpm for 5 min. The supernatants were used as the DNA template in all PCR. Detect 16 VFs of all *E. coli* isolates. These VFs represent the main categories of virulence determinants, including adhesins (*sfa, papC, sepA, etrA, aer, feaG, fsaA*, and *eaeA*), capsule synthesis (*rfc*), and toxins (*cnf1, hlyA, eltA, estA, exhA, stx1*, and *stx2*) (Table 1; Hartleib et al., 2003; Boerlin et al., 2005; Chapman et al., 2006; Cheng et al., 2006; Alabsi et al., 2014).

**TABLE 1:** List of 16 virulence genes detected in the present study, categorized based on their association with *E. coli* pathotypes.

**Pathotype**	**Virulence factors**	**Function**	**Primer sequence (5′–3′)**		**Annealing (°C)**	**Fragment size (bp)**	**References**
			**Forward**	**Reverse**			
EXPEC	*sfa*	S fimbriae (sialic acid-specific)	CTCCGGAGAACTGGGTGC ATCTTAC	CGGAGGAGTAATTACAAACC TGGCA	64	410	Chapman et al., 2006
	*cnf1*	Cytotoxic necrotizing factor 1	AAGATGGAGTTTCCTA TGCAGGAG	CATTCAGAGTCCTGCCC TCATTATT	63	498	Alabsi et al., 2014
	*papC*	Pilus associated with pyelonephritis	GTGGCAGTATGAGTAA TGACCGTTA	ATATCCTTTCTGCAGG GATGCAATA	64	200	Chapman et al., 2006
	*hlyA*	α-Hemolysin	AACAAGGATAAGCACT GTTCTGGC	ACCATATAAGCGGTCA TTCCCGTCA	63	1177	Chapman et al., 2006
	*rfc*	Lipopolysaccharide synthesis	ATCCATCAGGAGGGG ACTGGA	AACCATACCAACCAATGCGAG	63	788	Chapman et al., 2006
	*sepA*	Secreted serine protease of the auto-transporter family	TAAAACCCGCCGCCTGAGTA	TGCCGGTGAACAGGAGGTTT	62	611	Boerlin et al., 2005
EAEC	*etrA*	Component of ETT2 type III secretion system	CTTCTTCCTAACGAAACTA TCATTA	TGACATATCAACTTTC TCTTACGC	55	913	Hartleib et al., 2003
EIEC	*aer*	Aerobatin	TACCGGATTGTCATATG CAGACCGT	AATATCTTCCTCCAG TCCGGAGAAG	60	602	Alabsi et al., 2014
ETEC	*faeG*	F4 fimbrial adhesion	GAATCTGTCCGAGAATATCA	GTTGGTACAGGTCTTAATGG	55^b^	499	Chapman et al., 2006
	*fasA*	Fimbrial adhesion	GTAACTCCACCGTTTGTATC	AAGTTACTGCCAGTCTATGC	62	409	Boerlin et al., 2005
	*eltA*	Heat-labile enterotoxin	GGCGTTACTATCCTCTCTAT	TGGTCTCGGTCAGATATGT	55	272	Chapman et al., 2006
	*estA*	Heat-stable enterotoxin	CAACTGAATCACTTGACTCTT	TTAATAACATCCAGCACAGG	55	158	Boerlin et al., 2005
EPEC	*eaeA* ^a^	Intimin/attaching and effacing	GACCCGGCACAAGCATAAGC	CCACCTGCAGCAACAAGAGG	63	384	Chapman et al., 2006
	*exhA* ^a^	Enterohemolysin	GCATCATCAAGCGTACGTTCC	AATGAGCCAAGCTGG TTAAGCT	63	534	Chapman et al., 2006
EHEC	*eaeA* ^a^	Intimin/attaching and effacing	Same as above				
	*stx1*	Shiga-toxin-I	TGTCGCATAGTGGAACCTCA	TGCGCACTGAGAAGAAGAGA	58	655	Cheng et al., 2006
	*stx2*	Shiga-toxin-II	CCATGACAACGGA CAGCAGTT	TGTCGCCGATTATC TGACATTC	58	477	Cheng et al., 2006
	*exhA*	Enterohemolysin	Same as above				

*^a^Indicated genes shared by more than one E. coli pathotype.*

*^b^Add 3 s to the annealing time for each next cycle.*

### Phylogenetic Grouping

*E. coli* strains were assigned to one of the phylogroups A, B1, B2, C, D, E, or F by quadruplex PCR (c*huA*, *yjaA*, *arpA*, and DNA fragment TspE4.C2) using a previously described protocol (Clermont et al., 2013). All testing was performed with positive and negative controls. The PCR results were interpreted as previously described (Clermont et al., 2013).

### Biofilm Formation

The biofilm formation of each *E. coli* isolate was detected using the microtiter plate (MTP) method as described previously by Arezoo with slight modifications (Noie Oskouie et al., 2019). Briefly, 10 μL of each overnight culture was added in 1 mL LB broth for the production of biofilm. The suspension was adjusted to approximately 0.5 McFarland Standard (absorbance value of 0.08–0.1 at OD625 nm) and 180 μL was then transferred in triplicate into 96-wells without shaking at 37°C for 36 h. A control strain of *E. coli* (ATCC 25922, China Institute of Veterinary Drug Control) was used as a positive in each assay. After incubation, the microtiter plates were washed three times with phosphate buffered saline (PBS, PH 7.4) to remove all non-adherent bacteria. Methanol (200 μL) was added to each well for 15–20 min for fixing the attached bacteria. After removal of excess liquid, wells were washed three times with PBS before being stained for 5 min with 200 μL of 1% crystal violet at room temperature. After staining with crystal violet, the extra stain was rinsed off by placing the microtiter plate under running tap water and then air-dried at room temperature. The crystal violet was solubilized with 160 μL of 33% glacial acetic acid and then measured with an automated microplate reader (BioTek, Instruments, Inc. of Winooski, VT, United States) at OD570 nm measurement.

All strains were classified into four categories according to OD of bacterial, namely, non-adherent (–), weakly (+), moderately (++), or strongly adherent (+++). The cut-off ODc was defined for the microplate test as mean OD plus three standard deviations. In the current study were considered OD ≤ ODc, non-adherent, ODc<OD ≤ 2 ODc, weakly, 2 ODc<OD ≤ 4 ODc, moderately, and strongly adherent 4 ODc<OD as described previously (Stepanovic et al., 2000). The biofilm formation of each isolate was tested in triplicate (9 observations per isolate) and the results were averaged.

### Statistical Analysis

All analyses were conducted using SPSS for Windows Release 21 (SPSS Inc., United States) and GraphPad Prism 9 (GraphPad Software Inc., San Diego, CA, United States). Comparisons of association between antibiotic-resistant phenotypes, resistance genes, virulence genes, phylogenetic groups, and biofilm formation for *E. coli* isolates from different feeding models of wool sheep were separately by using the Pearson’s Chi-squared exact test. For all comparisons, *P* < 0.05 was considered statistically significant. Continuous variables were summarized as means and 95% confidence interval (CI). An association was considered positive when the two genes were found at the same time, otherwise, is was negative.

## Results

### Phenotypic Resistance

Overall, 500 *E. coli* isolates comprising 316 from intensive farming wool sheep [Ningxia, 25 farms (*n* = 266); Shaanxi, 5 farms (*n* = 50)] and 184 from free-range wool sheep [Qinghai, 18 farms (*n* = 134), Inner Mongolian, 6 farms (*n* = 50)] were obtained from 575 rectal swabs. The susceptibility of these isolates to 12 antimicrobials is shown in Table 2. The highest resistance rates were to sulfisoxazole (82.4%), florfenicol (72.0%), and tetracyclines (53.2%), followed by mequindox (46.8%), ampicillin (43.6%), spectinomycin (38.6%). enrofloxacin (34.2%), ceftiofur (21%), gentamycin (20.4%), ceftazidime (17.8%), and polymyxin B (7.8%). No resistance was found to meropenem in different groups of wool sheep. Notably, only one *E. coli* strains (0.2%) were susceptible to all the antibiotics, 122 (24.4%) were resistant to one or two antibiotics, and 377 (75.4%) were multiresistant (resistant to three or more antibiotics). Co-resistance involving sulfisoxazole, florfenicol, and tetracyclines were the most prevalent resistance detected. However, a few isolates were found to be multi-drug resistant to polymyxin B, ceftazidime, and gentamycin as well. Ninety-six resistance patterns were observed, of which SF-SPT-MEQ-AMP-TET-FFC was the most common.

**TABLE 2:** MIC distribution of *E. coli* (*N* = 500) and prevalence of resistance by isolation source.

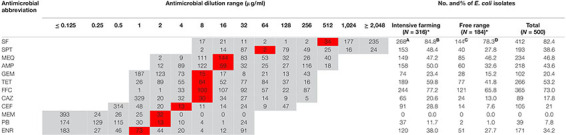

*SF, Sulfisoxazole; SPT, Spectinomycin; MEQ, Mequindox; AMP, Ampicillin; GEM, Gentamycin; TET, Tetracyclines; FFC, Florfenicol; CAZ, Ceftazidime; CEF, Ceftiofur; MEM, Meropenem; PB, Polymyxin B; ENR, Enrofloxacin.*

Gray and red colors indicate applied dilution ranges and breakpoints, respectively.

* Statistically significant difference (P < 0.05) between intensive farming and free-range (expect mequindox).

*A and C define intensive farming (Ningxia and Shaanxi) and free-range (Qinghai and Inner Mongolia) isolates showing microbial resistance, that is, MIC value higher than breakpoint.*

*B and D represent the percentage of drug-resistant strains in intensive farming and free-range, respectively.*

Wool sheep *E. coli* from two different farming systems have different phenotypic resistance to 11 antibiotics (except meropenem). Phenotypic resistance to 11 antibiotics (except meropenem) varied between the wool sheep from two different farming systems. Sulfisoxazole resistance was identified in 268 (84.8%) of intensive farming, 144 (78.3%) of the free-range wool sheep isolates. Whilst resistance to florfenicol was observed in 244 (77.2%) of intensive farming, 121 (65.8%) of the free-range wool sheep. The proportion of MDR *E. coli* was higher in intensive farming compared to free-range sheep. Surprisingly, 12 (2.4%) of the isolates were resistant to all antimicrobials other than meropenem, and they are derived from intensive farming. There were significant differences (*P* < 0.05) found between the occurrence of various resistance in wool sheep from two different farming systems (expect mequindox). In summary, the strains from free-range sheep had fewer antibiotic resistance than strains of intensive farming sheep.

### Prevalence of Virulence Genes and Occurrence of *E. coli* Pathotypes

Fifteen out of sixteen virulence genes were detected, namely, *etrA* (80.2%), *exhA* (68%), *eaeA* (39.6%), *fasA* (31.2%), *aer* (28%), *stx2* (22%), *stx1* (7.4%), *hlyA* (6.6%), *sepA* (6.2%), *sfa* (5.4%), *estA* (5.2%), *faeG* (4.6%), *papC* (3.8%), *eltA* (2.8%), and *cnf1* (2.4%). However, none of the isolates were positive to *rfc*, and the *etrA* was most prevalent among the virulence genes. Furthermore, only 26 strains of *E. coli* did not contain any of the 16 virulence genes. Overall, the majority of the *E. coli* isolates carried the combinations of virulence genes, associated with both IPEC and ExPEC pathotypes. Most of the *E. coli* isolates carried several different virulence genes, which are associated with both EAEC (80.2%) and EHEC (75%) pathotypes. In addition, 73.2, 36.8, and 28% of the isolates were positive for virulence genes associated with EPEC, ETEC, and EIEC pathotypes, respectively. Meanwhile, 20.4% of strains of our collection were considered as ExPEC pathotype (Table 3). In addition, the EAEC (86.4%), ETEC (40.8%), and ExPEC (22.3%) pathotypes were mainly observed in intensive farming. In contrast, EHEC (75%), EPEC (73.2%), and EIEC (29.7%) pathotypes were mainly carried out in free-range. There were significant differences (*P* < 0.05) found among EAEC, EHEC, and EPEC between intensive farming and free-range sheep. In summary, the strains from free-range sheep had fewer virulence genes than strains from intensive farming sheep.

**TABLE 3:** The percentage prevalence of the different pathotypes between intensive farming and free-range sheep.

**Pathotypes**	**Virulence factors**	**Free-range (*n* = 184)**	**No. and (%)**	**Intensive farming (*n* = 316)**	**No. and (%)**	**Total (*n* = 500)**	**Percentages (%)**
EAEC	*etrA*	159	159 (86.4)	242	242 (76.6)	401	80.2
EHEC	*eaeA*	57	122 (66.3)	141	253 (80.1)	375	75.0
	*stx1*	17		20			
	*stx2*	45		65			
	*exhA*	110		230			
EPEC	*eaeA*	57	116 (63.0)	141	250 (79.1)	366	73.2
	*exhA*	110		230			
ETEC	*faeG*	6	75 (40.8)	17	109 (34.5)	184	36.8
	*fasA*	66		90			
	*eltA*	2		12			
	*estA*	12		14			
EIEC	*aer*	46	46 (25.0)	94	94 (29.7)	140	28.0
ExPEC	*sfa*	15	41 (22.3)	12	61 (19.3)	102	20.4
	*cnf1*	2		10			
	*papC*	7		12			
	*hlyA*	15		18			
	*rfc*	0		0			
	*sepA*	12		19			

### Associations Between Virulence Genes and Resistance Phenotypes

We observed a number of virulence-associated genes of both IPEC and ExPEC categories in diarrheic wool sheep. The *etrA* and *hlyA* virulence genes were the most abundant, which are linked with ExPEC and IPEC pathotypes, respectively. There were significant statistical differences(*P* < 0.05) between the occurrence of different virulence genes (Supplementary Table 1). It can be seen from the data in Supplementary Table 1 that positive associations are more common than negative associations. Within the isolates, there was a marked association of *etrA*, *sfa*, *papc*, *aer*, *fasA*, *exhA*, and *stx2*. Out of the several different associations in the strains, the strongest correlation between the *etrA* and *papC* gene (*P* < 0.001, OR = 455.68) was found. Such findings might be an explanation for the high levels of virulence genes in *E. coli* of diarrhea wool sheep.

In this study, a statistically significant (*P* < 0.05) association between virulence genes and resistance phenotypes among all the *E. coli* isolates was tested (Supplementary Table 2). Surprisingly, 146 out of 165 observations had epidemiological relevance (*P* < 0.05). Overall, a positive association was more common than a negative one between virulence genes and resistance phenotypes. In addition, ExPEC-associated P fimbriae gene (*papC*), ETEC-associated heat-labile enterotoxin gene (*eltA*), and heat-stable enterotoxin gene (*estA*) were correlated with all antibiotics other than meropenem. A more detailed analysis displayed associations between the resistance/susceptibility phenotypes with potential virulence genes. All virulence-associated genes were significantly related to certain antibiotic resistance (including sulfisoxazole, tetracyclines, and ceftazidime). Except for sulfisoxazole, all antibiotics were negatively correlated with *etrA* and *exhA* genes. However, we observed the strongest association between ETEC-associated heat-labile enterotoxin (*eltA*) and sulfisoxazole (*P* < 0.001, OR = 877).

Overall, there was no direct association between all virulence factors and resistance phenotypes in *E. coli* of wool sheep. However, EAEC associated component of the ETT2 type III secretion system gene (*etrA*) was negatively correlated with resistance phenotypes (*P* < 0.05). Conversely, the EHEC- and EPEC-associated virulence genes were positively correlated with drug-resistant phenotypes (*P* < 0.05). Apart from this, ETEC- and ExPEC-associated virulence genes were also negatively associated with resistance phenotypes. The EIEC-associated aerobatin gene (*aer*) was positively correlated with resistance phenotypes.

### Occurrence of Phylogroups and Biofilm Formation

**FIGURE 2:**
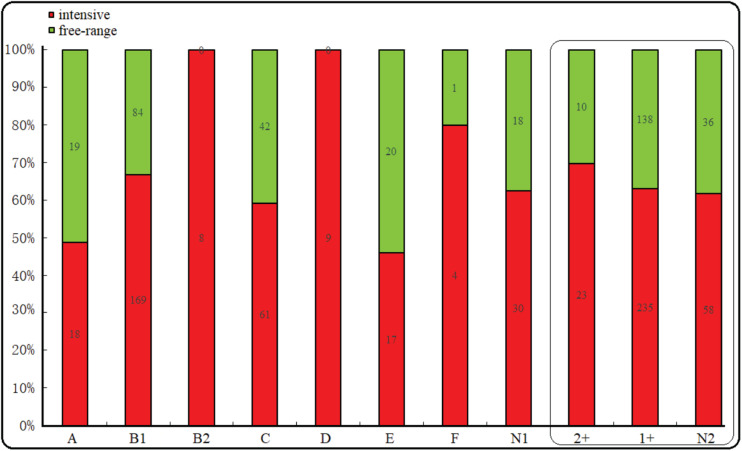
Phylogenetic and biofilm formation in the wool sheep *E. c*oli from two different farming systems (n = 500). The box inside represented the result of the biofilm formation. N1 indicated unclassified phylogroups. Based on the OD the strain’s biofilm formation was determined as: no biofilm formation N2, weak (1+) and moderate (2+) biofilm formation. Red and green colors indicated intensive and free-range, respectively.

The majority of the *E. coli* isolates belonged to phylogroup B1 (50.6%), followed by phylogroup C (20.6%), A (7.4%), E (7.4%), D (5.8%), B2 (1.6%), F (1%), and unclassified (9.6%). However, regardless of whether they were free-range or intensive farming sheep, B1 was the most prevalent phylogroup. Interestingly, phylogroup B2 and D were all distributed in intensive farms. In addition, phylogroup A, E, and C were more common in free-range. Among the 500 *E. coli* isolates from the diarrhea of wool sheep, 33 isolates (6.6%), 373 isolates (74.6%), and 94 (18.8%) showed moderate, weak, and no connection biofilm formation ability, respectively (Figure 2). Significant differences, but no linking biofilm formation ability was found between free-range and intensive. However, we found that the biofilm formation ability of intensive farming was better than free-range.

### Distribution of Virulence Profile in Relation to Phylogenetic Grouping and Biofilm Formation

In intensive farmed sheep, PCR analysis of 316 isolates revealed 53.48% of the isolates belong to phylogenetic B1, followed by group C (19.30%), A (5.70%), E (5.38%), D (2.85%), B2 (2.53%), F (1.27%), and non-phylogenetic (9.49%). In free-range sheep, PCR analysis of 184 isolates revealed that 45.65% of the isolates belong to phylogenetic B1, followed by group C (22.83%), A (10.33%), E (10.87%), D (0.00%), B2 (0.00%), F (0.54%), and non-phylogenetic (9.78%). Those from the intensive farms and free-range wool sheep *E. coli* were predominantly from phylogenetic groups B1 and C. The distribution of the 500 *E. coli* isolates in relation to virulence genes and phylogenetic groups from the different farming systems of wool sheep revealed that out of the 474 isolates containing virulence factors, 242 belonged to group B1, 100 to group C, 37 to group A, 37 to group E, 9 to group D, 6 to B2, and 5 to group F (Table 4). Within the isolates, *etrA*, *exhA*, and *eaeA* genes were the commonly detected genes, whereas *rfc* gene was not identified in all *E. coli* isolates. Interestingly, *etrA* + *exhA* + *eaeA* + *cnf1* (n = 2), *exhA* + *eaeA* + *stx2* + *hlyA* (*n* = 2) and *etrA* + *exhA* + *eaeA* + *fasA* + *eltA* (*n* = 2) were found only in intensive farming, whereas *fasA* + *stx1* (*n* = 1) was only in free-range wool sheep. For various feeding modes of wool sheep, it was found that intensive isolates contained more tested virulence genes. The capacity of biofilm formation was compared between various virulence profiles, where there was no significant correlation between them; however, the moderate had related to *etrA*, *exhA*, and *eaeA* gene. Notably, the *fasA* gene may combine with other genes to reduce the formation of biofilm.

**TABLE 4:** Distribution of virulence genes in relation to phylogenetic grouping.

**Prevalence of Vfs**	**Free-range wool sheep (*n* = 184)**												**Intensive farming (*n* = 316)**												**Total (%) (*n* = 500)**
	**Phylogenetic group**									**Biofilm formation**			**Phylogenetic group**									**Biofilm formation**			
	**A**	**B1**	**B2**	**C**	**D**	**E**	**F**	**N**	**Total**	**2 +**	**1 +**	**N**	**A**	**B1**	**B2**	**C**	**D**	**E**	**F**	**N**	**Total**	**2 +**	**1 +**	**N**	
*etrA*	2	16	–	26	–	11	–	2	57	2	41	14	–	32	1	9	2	1	–	4	49	2	41	6	106(21.2)
*exhA*	2	7	–	–	–	–	–	2	11	2	8	1	3	19	2	7	1	3	–	3	38	4	26	8	49(9.8)
*eaeA*	1	–	–	–	–	–	–	–	1	1	–	–	–	1	–	–	1	1	–	2	5	3	2	–	6(1.2)
*fasA*, *stx1*	–	–	–	–	–	–	–	1	1	–	1	–	–	–	–	–	–	–	–	–	0	–	–	–	1(0.2)
*sepA*, *estA*	–	–	–	–	–	–	–	–	0	–	–	–	–	–	–	–	–	–	–	1	1	–		1	1(0.2)
*hlyA*, *eaeA*	–	–	–	1	–	–	–	–	1	–	1	–	–	1	–	–	–	–	–	–	1	–	1	–	2(0.4)
*etrA*, *exhA*	9	38	–	6	–	5	–	2	60	2	45	13	8	60	–	20	2	6	2	4	102	7	74	21	162(32.4)
*etrA*, *exhA*, *sfa*	1	1	–	–	–	–	–	1	3	–	3	–	–	4	–	–	–	–	–	–	4	–	4	–	7(1.4)
*etrA*, *exhA*, *papC*	–	–	–	–	–	–	–	1	1	1	–	–	–	2	–	1	–	–	–	–	3	–	3	–	4(0.8)
*etrA*, *exhA*, *aer*	1	2	–	1	–	–	–	–	4	1	2	1	3	8	1	2	–	3	1	1	19	1	15	3	23(4.6)
*etrA*, *exhA*, *faeG*	–	1	–	–	–	–	1		2	–	2	–	–	–	–	3	–	–	–	–	3	–	2	1	5(1.0)
*etrA*, *exhA*, *eaeA*	2	8	–	1	–	3	–	1	15	1	9	5	4	13	1	10	1	2	–	6	37	4	28	5	52(10.4)
*etrA*, *exhA*, *aer*, *stx2*	1	4	–	2	–	–	–	–	7	–	7	–	–	3	–	–	–	–	–	1	4	1	2	1	11(2.2)
*etrA*, *exhA*, *eaeA*, *cnf1*	–	–	–	–	–	–	–	–	0	–	–	–	–	–	–	1	–	–	–	1	2	–	2	–	2(0.4)
*etrA*, *exhA*, *eaeA*, *fasA*	–	4	–	–	–	–	–	2	6	–	5	1	–	9	–	3	–	–	–	–	12	1	9	2	18(3.6)
*etrA*, *sepA*, *estA*, *fasA*	–	–	–	2	–	1	–	–	3	–	3	–	–	2	–	1	–	–	–	–	3	–	3	–	6(1.2)
*exhA*, *eaeA*, *stx2*, *hlyA*	–	–	–	–	–	–	–	–	0	–		–	–	–	–		1	–	1	–	2	–	2	–	2(0.4)
*etrA*, *exhA*, *eaeA*, *fasA*, *eltA*	–	–	–	–	–	–	–	–	0	–	–		–	–	–	1	–	1	–	–	2	–	1	1	2(0.4)
*etrA*, *exhA*, *eaeA*, *fasA*, *stx1*	–	1	–	–	–	–	–	–	1	–	1	–	–	1	–	1	–	–	–	–	2	–	2		3(0.6)
*sfa*(1)/*hlyA*(1)/*sepA*(2)/*aer*(4)/*faeG*(1)/*fasA(*2)/*stx2*(1)	–	1	–	2	–	–	–	1	4	–	4	–	–	4	1	–	1	–	–	2	8	–	6	2	12(2.4)
Total no. of isolates with Vfs	19	83	0	41	0	20	1	13	177	10	132	35	18	159	6	59	9	17	4	25	297	23	223	51	474(94.8)
No of isolates without Vfs	–	1	–	1	–	–	–	5	7	–	6	1	–	10	2	2	–	–	–	5	19	–	12	7	26(5.3)

*–, indicates none of the isolates were positive*.

*N, indicates that isolate strains were not assigned to any phylogenetic group, and biofilm formation.*

## Discussion

Worldwide, pathogenic *E. coli* isolates have been increasingly reported from animal, animal-derived foods, and humans (Dorado-García et al., 2018; Wang et al., 2020). Humans could obtain resistance/virulence genes from *E. coli* via the food chain (Madec et al., 2015). In this study, we observed a higher proportion of multidrug-resistant strains with virulence genes in wool sheep. This appears to be the first study to systematically compare antimicrobial resistance, virulence, biofilm, and phylogenetic groups in *E. coli* isolates from intensive and free-range wool sheep in China. During the period of investigation, a total of 500 *E. coli* strains were isolated, of which, 316 were isolated from intensive farming sheep (Ningxia and Shaanxi) and 184 were isolated from free-range sheep (Qinghai and Inner Mongolia). *E. coli* isolated from free-range sheep have a lower prevalence than intensive farming wool sheep. The low level of resistance and virulence in the free-range sheep were not surprising as antibiotic treatment is rare in these areas. In addition, the possible lower feeder density coupled with *ad libitum* green grass in free farming might explain lower levels of resistance and virulence when compared to intensive farming. These data can be compared to reports on other animals since studies of resistance in wool sheep are still insufficient.

Among all *E. coli* isolates strains, 75% were resistant to MDR, whereas 2.4% of the strains were resistant to all antimicrobials other than meropenem. Furthermore, only one *strain* was sensitive to all antibiotics. The *E. coli* isolates from different provinces have different resistance patterns. Specifically, the prevalence of MDR infection rate in *E. coli* of wool sheep origin in Inner Mongolia was significantly lower than in other provinces. In addition, we found a higher resistance rate to several old drugs, including sulfisoxazole (82.4%), florfenicol (72.0%), and tetracyclines (53.2%), consistent with previous reports (Zhang et al., 2017; Ma et al., 2021). The predominance of sulfisoxazole resistance strains was similar to the findings of Zhang et al. (2017), who detected sulfisoxazole resistance in 6,276 out of 7,568 *E. coli* isolates of chicken. Likewise, the predominance of florfenicol resistance strains was similar to Ma et al. (2021) The report stated that he detected florfenicol resistance in 36 out of 55 *E. coli* strains. Moreover, *E.coli* resistance to tetracyclines has been widely detected in pigs, chickens, and soil in China (Sun et al., 2019). Most notably, similar levels of *E. coli* resistance were observed in animals and the environment. This could be due to empiric antibiotic treatment use, indiscriminate, and overutilization of antibiotics in human medicine and livestock feed in China (Kümmerer, 2009). The long history of using these drugs in animals (tetracyclines and sulfonamides in the 1960s; florfenicol in the 1980s) may cause high prevalence resistance to these drugs. We detected high rates of AMR, with 75% of isolates displaying resistance to ≥ 3 antibiotic classes. By comparing the two different farming systems, intensive farming isolates showed higher levels of resistance to 11 tested antibiotic agents. The most common co-resistant phenotype observed was sulfisoxazole and florfenicol. Fortunately, we observed no resistance against meropenem in wool sheep from two different farming systems. Meropenem is considered as the last–resort drug for severe bacterial infections (Koeck et al., 2018). Thus, the phenomenon of antibiotic resistance has become a global public health concern. The problem of antibiotic resistance is worsening and is not optimistic in China. Therefore, the government needs new antibiotic treatment guidelines, which necessitate a strong resistance surveillance program in the western region of China.

As a direct pathogenic factor, the risk virulence factor of *E. coli* has drawn much attention from researchers. In this study, 15 virulence genes were identified in all *E. coli* isolate strains, indicating that diarrhea sheep harbored abundant and diverse virulence factors. Overall, our results showed that a significant proportion (23.6%) of *E. coli* from diarrheic sheep were possible simultaneously to IPEC and ExPEC pathogenic types. It is particularly worrisome that a significant fraction of *E. coli* isolates were positive for at least two of the five intestinal pathogenic. In this study, *etrA* (80.2%), and *exhA* (68%) genes were the most prevalent. This is consistent with previous studies identifying EAEC and EPEC pathotypes to have emerged as an increasingly important cause of diarrhea worldwide (Bolick et al., 2013; Hernandes et al., 2020; Ranganathan et al., 2020), and those showing a high frequency positive EAEC (80.2%) and EHEC (75%) strains in this study. Our research results showed that 94.8% of strains showed at least one virulence gene, nevertheless, the occurrence of virulence genes in the *E. coli* strain does not indicate its pathogenicity property (Rehman et al., 2017). Therefore, further studies, including additional animal model studies or bioinformatics approaches, are necessary to demonstrate the pathogenicity of observed virulence genes. All isolates were positive for the IPEC associated virulence genes, but negative for ExPEC associated virulence genes (*rfc*). In previous reports, such results have not been commonly described as animal origin *E. coli* isolates (Obeng et al., 2012; Osugui et al., 2014). Additionally, we also observed that a high frequency of *E. coli* isolates harbored *eaeA* (39.6%) and *fasA* (31.2%) virulence genes. The exact clinical significance of this funding is not clear. However, the *eaeA* gene and *fasA* gene may play a major role in adherence/colonization, causing attaching/effacing lesions (Rehman et al., 2017; Stromberg et al., 2020). The emergence of virulence gene patterns observed in the current study might be due to horizontal gene transfer mediating transfer virulence factors (Håkonsholm et al., 2020).

In our study, we also observed an association between the drug-resistant phenotype and virulence factors. In brief, negative correlations were more common. We observed the strongest association between ETEC associated toxin gene (*eltA*) and sulfisoxazole (*P* < 0.001, OR = 877). We identified a positive association between EAEC associated adhesion gene (*etrA*) and 10 antibiotics other than sulfisoxazole. This implies the possible selective advantage of *E. coli* strains harboring resistance and virulence genes. This could spell doom for current efforts to control infections with *E. coli* because animal husbandry would be overwhelmed by more virulent strains and increased drug resistance. Taken together, our findings suggest that the association of resistance and virulence genes might be multiple strain-specific. Moreover, we only examined the phenotypic profile in this study. Therefore, further studies are required to confirm this association between drug resistance and virulence of *E. coli* to forestall a potential health hazard.

As previously suggested, all *E. coli* strains can be assigned to one of the seven well-recognized phylogenetic groups, namely A, B1, B2, C, D, E, and F, as previously suggested (Clermont et al., 2013). It has been reported that *E. coli* phylogenetic groups A and B1 are typically commensal strains, whereas the B2 and D groups are mainly urinary tract infection strains (Song et al., 2020). In this study, based on the phylogenetic analysis, we found that antibiotic resistance was associated with virulence among *E. coli* strains. The majority of the *E. coli* isolates belonged to group B1 (50.6%) and the remaining to phylogroups A, B2, C, D, or F. Moreover, the *E. coli* component of the ETT2 type III secretion system gene (*etrA*) was the most common gene observed in phylogroup B1. The combination of phylogroup B1 and *etrA* gene might play an important role in diarrheic infections. Notably, phylogroups B2 and D only appear in intensive farming. This finding coincides with phenotypic resistance and virulence genes among *E. coli* strains. In addition, the diversity of this phylogroup in intensive farming sheep might be related to the widespread use of antibiotics, physiognomy, and climate compared to free-range sheep. Furthermore, our findings were consistent with previous studies of diarrheagenic and commensal strains (Adamus-Bialek et al., 2009). Our biofilm formation results were different from those reported in previous literature (Tajbakhsh et al., 2016). Comparing the wool sheep origin *E. coli* from two different farming systems, there was no significant difference in biofilm formation. However, the biofilm formation might be related to virulence genes *etrA, exhA*, and *eaeA*.

## Conclusion

This is the first systematical comparison of antimicrobial resistance, virulence factors, phylogenetic grouping, and biofilm formation among *E. coli* isolates from wool sheep in intensive and free-range farming systems in China. In the present study, we observed a higher proportion of multidrug-resistant *E. coli* with virulence factors in sheep suffering from diarrhea and determined the correlations among virulence genes and resistance phenotypes. These results reveal that close monitoring of the virulence and antimicrobial resistance of food animal production is essential and that it could reduce the potential public health risks in both animal and human medicines.

## Data Availability Statement

The original contributions presented in the study are included in the article/Supplementary Material, further inquiries can be directed to the corresponding author/s.

## Ethics Statement

The animal study was reviewed and approved by Northwest A&F University Ethics Committee.

## Author Contributions

XLZ, JW, ZQY, and XLW conceived and designed the project. XLZ, YYL, QFX, XDB, BW, XYW, and RYD performed the experiments. XLZ and HHS analyzed the data. XLZ, FEAA, and HJL wrote and revised the manuscript. All authors read and approved the final manuscript.

## Conflict of Interest

The authors declare that the research was conducted in the absence of any commercial or financial relationships that could be construed as a potential conflict of interest.

## Publisher’s Note

All claims expressed in this article are solely those of the authors and do not necessarily represent those of their affiliated organizations, or those of the publisher, the editors and the reviewers. Any product that may be evaluated in this article, or claim that may be made by its manufacturer, is not guaranteed or endorsed by the publisher.

## Footnotes

1
https://www.cdc.gov/narms/antibiotics-tested.html
