# Supplementary material for: Comparison of Antimicrobial Resistance, Virulence Genes, Phylogroups, and Biofilm Formation of Escherichia coli Isolated From Intensive Farming and Free-Range Sheep
Source: Front Microbiol. 2021 Jul 30;12:699927. doi: 10.3389/fmicb.2021.699927 (PMC8362090; doi:10.3389/fmicb.2021.699927)
Supplement: Supplementary file 1 [file Table_1.docx]

Table S1 The associations between virulence genes among *E. coli* (N=500)

| Virulence gene (n) | Associations of gene (OR, 95% confidence interval)^a^ | | | | | | | | | | | | | | |
| --- | --- | --- | --- | --- | --- | --- | --- | --- | --- | --- | --- | --- | --- | --- | --- |
|  | *sfa* | *cnf1* | *papC* | *hlyA* | *sepA* | *etrA* | *aer* | *faeG* | *fasA* | *eltA* | *estA* | *eaeA* | *exhA* | *stx1* | *stx2* |
| *sfa* (27) | NS | – | 3.98  (1.40-11.33) | 8.76  (3.60-21.31) | – | 140.15  (39.70-494.74) | 5.01  (1.87-13.43) | – | 16.27  (6.96-38.01) | – | – | 25.48  (10.78-60.22) | 218.98  (49.28-973.17) | – | 7.38  (2.96-18.37) |
| *cnf1* (12) | – | NS | 13.56  (3.26-56.46) | 8.13  (1.61-41.21) | – | 447.33  (53.39-3748.17) | 29.05  (8.06-104.75) | – | 13.56  (3.26-56.46) | – | – | 56.93  (15.79-205.30) | 447.33  (53.39-3748.17) | – | 20.33  (5.38-76.87) |
| *papC* (19) | 3.98  (1.40-11.33) | 13.56  (3.26-56.46) | NS | – | – | 455.68  (57.78-3593.92) | 28.13  (10.24-77.27) | – | 14.77  (5.23-41.73) | – | – | 22.78  (8.29-62.59) | 215.18  (46.35-998.95) | – | 9.04  (3.00-27.69) |
| *hlyA* (3*3)* | 8.76  (3.60-21.31) | 8.13  (1.61-41.21) | – | NS | – | 52.56  (21.24-130.09) | 5.31  (2.28-12.34) | – | 4.53  (1.90-10.82) | – | – | 24.77  (11.21-54.69) | 63.68  (24.57-165.07) | – | 16.98  (7.86-36.71) |
| *sepA* (31) | – | – | – | – | NS | 102.12  (33.62-310.24) | – | – | 10.93  (4.91-24.33) | – | 4.41  (1.76-11.04) | – | – | – | – |
| *etrA* (401*)* | 140.15  (39.70-494.74) | 447.33  (53.39-3748.17) | 455.68  (57.78-3593.92) | 52.56  (21.24-130.09) | 102.12  (33.62-310.24) | NS | 0.13  (0.09-0.17) | 0.08  (0.06-0.11) | 0.14  (0.11-0.19) | 0.01  (0.01-0.02) | 0.02  (0.01-0.03) | 0.18  (0.14-0.25) | 0.64  (0.47-0.87) | 0.02  (0.01-0.03) | 0.08  (0.06-0.11) |
| *aer* (140) | 5.01  (1.87-13.43) | 29.05  (8.06-104.75) | 28.13  (10.24-77.27) | 5.31  (2.28-12.34) | – | 0.13  (0.09-0.17) | NS | 0.24  (0.13-0.45) | 1.18  (0.79-1.77) | 0.10  (0.04-0.24) | 0.14  (0.63-0.30) | 2.43  (1.65-3.57) | 7.16  (4.69-10.93) | 0.22  (0.12-0.42) | – |
| *faeG* (23) | – | – | – | – | – | 0.08  (0.06-0.11) | 0.24  (0.13-0.45) | NS | 38.89  (14.97-101.02) | – | – | 13.33  (5.23-34.00) | 74.66  (25.47-218.87) | – | 7.32  (2.64-20.31) |
| *fasA* (156) | 16.27  (6.96-38.01) | 13.56  (3.26-56.46) | 14.77  (5.23-41.73) | 4.53  (1.90-10.82) | 10.93  (4.91-24.33) | 0.14  (0.11-0.19) | 1.18  (0.79-1.77) | 38.89  (14.97-101.02) | NS | 0.09  (0.04-0.20) | 0.15  (0.08-0.30) | – | 5.99  (4.01-8.94) | 0.26  (0.15-0.44) | – |
| *eltA* (14) | – | – | – | – | – | 0.01  (0.01-0.02) | 0.10  (0.04-0.24) | – | 0.09  (0.04-0.20) | NS | – | – | 5.79  (1.18-28.33) | – | 5.79  (1.18-28.33) |
| *estA* (26) | – | – | – | – | 4.41  (1.76-11.04) | 0.02  (0.01-0.03) | 0.14  (0.63-0.30) | – | 0.15  (0.08-0.30) | – | NS | 3.32  (1.06-10.32) | 3.32  (1.06-10.32) | – | – |
| *eaeA* (198) | 25.48  (10.78-60.22) | 56.93  (15.79-205.30) | 22.78  (8.29-62.59) | 24.77  (11.21-54.69) | – | 0.18  (0.14-0.25) | 2.43  (1.65-3.57) | 13.33  (5.23-34.00) | – | – | 3.32  (1.06-10.32) | NS | 10.09  (6.44-15.82) | 0.16  (0.10-0.27) | 0.63  (0.44-0.90) |
| *exhA* (340) | 218.98  (49.28-973.17) | 447.33  (53.39-3748.17) | 215.18  (46.35-998.95) | 63.68  (24.57-165.07) | – | 0.64  (0.47-0.87) | 7.16  (4.69-10.93) | 74.66  (25.47-218.87) | 5.99  (4.01-8.94) | 5.79  (1.18-28.33) | 3.32  (1.06-10.32) | 10.09  (6.44-15.82) | NS | 0.05  (0.04-0.08) | 0.20  (0.15-0.27) |
| *stx1* (37) | – | – | – | – | – | 0.02  (0.01-0.03) | 0.22  (0.12-0.42) | – | 0.26  (0.15-0.44) | – | – | 0.16  (0.10-0.27) | 0.05  (0.04-0.08) | NS | 4.64  (2.09-10.31) |
| *stx2* (110) | 7.38  (2.96-18.37) | 20.33  (5.38-76.87) | 9.04  (3.00-27.69) | 16.98  (7.86-36.71) | – | 0.08  (0.06-0.11) | – | 7.32  (2.64-20.31) | – | 5.79  (1.18-28.33) | – | 0.20  (0.15-0.27) | 0.20  (0.15-0.27) | 4.64  (2.09-10.31) | NS |

–, indicates no significant associations (*P*≥0.05), NS, no statistics were determined

a, Odds ratio (OR) for associations between virulence genes (95% confidence interval in parenthesis)
