# Supplementary material for: Comparison of Antimicrobial Resistance, Virulence Genes, Phylogroups, and Biofilm Formation of Escherichia coli Isolated From Intensive Farming and Free-Range Sheep
Source: Front Microbiol. 2021 Jul 30;12:699927. doi: 10.3389/fmicb.2021.699927 (PMC8362090; doi:10.3389/fmicb.2021.699927)
Supplement: Supplementary file 2 [file Table_2.docx]

Table S2 The associations between virulence genes and resistance phenotypes among *E. coli* (N=500)

| Virulence gene (n) | Associations of gene (OR and 95% confidence interval) ^a^ | | | | | | | | | | |
| --- | --- | --- | --- | --- | --- | --- | --- | --- | --- | --- | --- |
|  | No. SF | No. SPT | No. MEQ | No. AMP | No. GEM | No. TET | No. FFC | No. CAZ | No. MEM | No. PB | No. ENR |
| *sfa* (27) | 24 140.15  (39.70-494.73) | 09 8.76  (3.60-21.31) | 15 21.90  (9.33- 51.35) | 10 10.31  (4.31-4.65) | – | 13 16.27  (6.96-38.01) | 20 50.05  (19.47-128.66) | 05 3.98  (1.40-11.33) | 05 3.98  (1.40-11.33) | – | 14 18.87  (8.07-44.08) |
| *cnf1* (12) | 10 203.33  (40.13-1030.15) | 06 40.67  (11.440-144.56) | 09 122  (29.29-508.09) | 06 40.67  (11.440-144.56) | 01 3.70  (0.44-31.0) | 03 13.56  (3.26-56.46) | 08 81.33  (21.51-307.48) | 03 13.56  (3.26-56.46) | 05 29.05  (8.06-104.75) | – | 04 20.33  (5.38-76.87) |
| *papC* (19) | 16 135.02  (36.23-503.20) | 07 14.77  (5.23-41.73) | 08 18.41  (6.64-51.04) | 07 14.77  (5.23-41.73) | 03 4.75  (1.27-17.70) | 09 22.78  (8.29-62.59) | 13 54.85  (18.81-159.99) | 04 6.75  (2.05-22.29) | 05 9.04  (2.95-27.69) | 02 2.98  (0.64-13.81) | 07 14.77  (5.27-41.73) |
| *hlyA* (33) | 26 52.56  (21.24-130.09) | 08 4.53  (1.90-10.82) | 14 10.43  (4.80-22.64) | 08 4.53  (1.90-10.82) | – | 14 10.43  (4.80-22.64) | 19 19.21  (8.85-41.70) | 07 3.81  (1.54-9.43) | 09 5.31  (2.28-12.34) | – | 08 4.53  (1.90-10.82) |
| *sepA* (31) | 26 78.67  (28.26-219.0) | 18 20.95  (9.41-46.70) | 18 20.95  (9.41-46.70) | 22 37.0  (15.71-87.09) | 10 7.20  (3.12-16.62) | 21 31.77  (13.77-73.31) | 25 63.04  (24.09-165.0) | 09 6.19  (2.63-14.58) | 08 5.26  (2.18-12.72) | – | 17 18.37  (8.29-40.69) |
| *etrA* (401) | 363 2.36  (1.58-3.52) | 160 0.16  (0.12-0.22) | 194 0.23  (0.17-0.31) | 175 0.19  (0.14-0.26) | 84 0.07  (0.05-0.09) | 212 0.28  (0.21-0.37) | 275 0.54  (0.40-0.73) | 77 0.06  (0.04-0.08) | 90 0.07  (0.05-0.10) | 30 0.02  (0.01-0.03) | 135 0.13  (0.09-0.17) |
| *aer* (140) | 124 19.93  (11.43-34.75) | – | 69 2.50  (1.70-3.67) | 63 2.10  (1.43-3.10) | – | 73 0.20  (1.91-4.12) | 82 3.64  (2.46-5.36) | 25 0.56  (0.35-0.90) | – | 11 0.22  (0.12-0.49) | – |
| *faeG* (23) | 21 217.76  (48.13-985.30) | 11 19.01  (7.58-47.65) | 15 38.89  (14.97-101.02) | 07 9.07  (3.40-24.22) | 06 7.32  (2.64-20.31) | 11 19.01  (7.58-47.65) | 12 22.63  (9.03-56.71) | 05 5.76  (1.97-16.89) | 05 5.76  (1.97-16.89) | – | 09 13.33  (5.29-34.00) |
| *fasA* (156) | 143 24.26  (13.33-44.13) | – | 88 2.85  (1.97-4.13) | 62 1.45  (1.00-2.11) | 32 0.57  (0.37-0.88) | 85 2.64  (1.83-3.81) | 107 4.82  (3.27-7.09) | 33 0.59  (0.39-0.91) | 33 0.59  (0.39-0.91) | 9 0.14  (0.07-0.27) | 63 1.49  (1.03-2.17) |
| *eltA* (14) | 14 877.00  (49.86-15426.30) | 09 56.31  (16.70-190.00) | 08 41.71  (12.76-136.40) | 06 23.46  (7.18-76.72) | 02 5.21  (1.07-25.54) | 11 114.71  (28.77-457.38) | 10 78.21  (21.84-280.13) | 04 12.51  (3.49-44.82) | 06 23.46  (7.18-76.72) | 02 5.21  (1.07-25.54) | 05 17.38  (5.15-58.63) |
| *estA* (26) | 24 218.77  (49.03-976.09) | 22 100.27  (32.19-312.31) | 19 49.48  (19.09-128.24) | 21 76.57  (26.74-219.28) | 12 15.63  (6.57-37.16) | 20 60.77  (22.49-164.21) | 22 100.27  (32.19-312.31) | 11 13.37  (5.59-32.00) | 09 9.66  (3.93-23.72) | 06 5.47  (2.02-14.78) | 16 29.17  (12.06-70.55) |
| *eaeA* (198) | 177 12.86  (7.90-20.91) | – | – | – | 32 0.29  (0.19-0.45) | 108 1.83  (1.31-2.55) | 127 2.73  (1.94-3.84) | 29 0.26  (0.17-0.40) | 42 0.41  (0.28-0.60) | 8 0.06  (0.03-0.13) | 60 0.66  (0.47-0.94) |
| *exhA* (340) | 308 4.53  (3.01-6.82) | 117 0.15  (0.18-0.33) | 148 0.36  (0.27-0.48) | 122 0.26  (0.20-0.35) | 58 0.10  (0.07-0.13) | 177 0.51  (0.39-0.68) | – | 44 0.07  (0.48-0.10) | 63 0.11  (0.08-0.15) | 17 0.03  (0.02-0.04) | 90 0.17  (0.13-0.23) |
| *stx1* (37) | 32 80.09  (29.46-217.75) | 10 4.64  (2.08-10.31) | 18 11.90  (5.73-24.51) | 13 6.78  (3.19-14.40) | 08 3.45  (1.47-8.09) | 15 8.53  (4.08-17.83) | 22 18.35  (8.79-38.34) | 06 2.42  (0.95-6.18) | 08 3.45  (1.47-8.09) | – | 12 6.01  (2.80-12.91) |
| *stx2* (110) | 96 24.31  (13.35-44.27) | – | 48 2.75  (1.78-4.23) | 35 1.66  (1.05-2.60) | 10 0.36  (0.18-0.70) | 55 3.55  (2.31-5.45) | 67 5.52  (3.37-8.56) | 10 0.36  (0.18-0.70) | 16 0.60  (0.34-1.07) | 03 0.10  (0.30-0.32) | – |

–, indicates no significant associations (*P* ≥ 0.05).

a, Odds ratio (OR) for associations between virulence genes and all antimicrobials (95% confidence interval in parenthesis).
